# Supplementary material for: Intracellular pathogen effector reprograms host gene expression by inhibiting mRNA decay
Source: Nat Commun. 2025 Jul 12;16:6452. doi: 10.1038/s41467-025-61194-2 (PMC12255788; doi:10.1038/s41467-025-61194-2)
Supplement: Supplementary file 1 — Supplementary Information [file 41467_2025_61194_MOESM1_ESM.pdf]

SUPPLEMENTARY INFORMATION

Intracellular Pathogen Effector Reprograms Host Gene Expression By Inhibiting mRNA Decay

Yevgen Levdansky, Justin C. Deme, David J. Turner, Claire T. Piczak, Filip Pekovic, Anna L. Valkov, Sergey G. Tarasov, Susan M. Lea, Eugene Valkov

National Cancer Institute, National Institutes of Health, Frederick MD 21702, U.S.A.

Supplementary Table 1. Thermodynamic parameters of NOT7 and NOT8 interactions with PieF variants determined by isothermal titration calorimetry.

Protein	Ligand	K_d (nM)	ΔH (kcal mol^{-1})	-TΔS (kcal mol^{-1})	ΔG (kcal mol^{-1})	Molar Ratio
NOT7	PieF WT	24.6 ± 4.14	-15.7 ± 0.7	5.33 ± 0.8	-10.4 ± 0.1	0.740 ± 0.05
	PieF K124A	564 ± 194	-11.8 ± 0.9	3.29 ± 1.0	-8.54 ± 0.2	0.724 ± 0.06
	PieF K124R	94.8 ± 30.9	-14.2 ± 0.5	4.61 ± 0.4	-9.60 ± 0.2	0.702 ± 0.03
	PieF 5M	NI*	NI	NI	NI	NI
	PieF 3M	NI	NI	NI	NI	NI
	PieF 2M	438 ± 182	-12.4 ± 0.6	3.68 ± 0.8	-8.70 ± 0.2	0.675 ± 0.1
NOT8	PieF WT	23.9 ± 9.20	-23.3 ± 2.6	12.9 ± 2.8	-10.4 ± 0.2	0.421 ± 0.040

*NI = No observed interaction; thermodynamic parameters could not be determined

All values represent averages from three independent experiments, and error values represent standard deviations. Experiments that showed no interaction (NI) were repeated twice. Both macromolecules contained an N-terminal SUMO tag connected via a GGS linker and TEV protease cleavage site. All ligands used contained an N-terminal His_6-tag connected via an SSGTGSG linker and a TEV protease cleavage site.

Supplementary Table 2. Cryo-EM data collection, refinement, and validation statistics.

	NOT1:NOT7:PieF (EMD-47689) (PDB 9E7T)	NOT1:NOT8:PieF (EMD-47690) (PDB 9E7U)
**Data collection and processing**		
Magnification	165,000	165,000
Voltage (kV)	300	300
Electron exposure (e–/Å^2)	55.6	52.4
Defocus range (μm)	-2.5 to -0.5	-2.5 to -0.5
Pixel size (Å)	0.732	0.732
Symmetry imposed	C1	C1
Initial particle images (no.)	7,114,506	4,808,811
Final particle images (no.)	417,581	200,675
Map resolution (Å)	2.8	3.5
FSC threshold	0.143	0.143
Map resolution range (Å)	2.3-41.0	2.9-49.9
**Refinement**		
Initial model used (PDB code)	none	none
Model resolution (Å)	2.9	3.6
FSC threshold	0.5	0.5
Map sharpening *B* factor (Å^2)	-108.3	deepEMhancer
Model composition		
Non-hydrogen atoms	4842	4867
Protein residues	597	601
Ligands	Mg:1	Mg:1
B factors (Å^2)		
Protein	36.70	47.88
Ligand	28.13	39.31
R.m.s. deviations		
Bond lengths (Å)	0.003	0.005
Bond angles (°)	0.454	0.652
**Validation**	1.30	2.24
MolProbity score	3.72	6.48
Clashscore	1.11	4.38
Poor rotamers (%)		
Ramachandran plot		
Favored (%)	97.45	94.10
Allowed (%)	2.55	5.90
Disallowed (%)	0.00	0.00
CC (mask)	0.86	0.76

Supplementary Table 3. DNA constructs.

All constructs were generated using the Gibson assembly method.

Plasmid	Protein	Residues	Tag/ Protease site	Comments
**pnEK-vH-PieF**	His_6-PieF	1-125	His_6 (N) / TEV	Amplified from synthetic PieF gene (provided by GenScript) and inserted into pnEK-vH. The original protein sequence is from NCBI NP_001309019.1
**pnYC-vS-PieF**	StrepII-PieF	1-125	StrepII (N) / TEV	Amplified from synthetic PieF gene (provided by GenScript) and inserted into pnYC-vS.
**pnEK-vH-PieF_K124A**	His_6-PieF^{K124A}	1-125	His_6 (N) / TEV	K124A mutation introduced in reverse primer; amplified from wildtype plasmid.
**pnEK-vH-PieF_K124R**	His_6-PieF^{K124R}	1-125	His_6 (N) / TEV	K124R mutation introduced in reverse primer; amplified from wildtype plasmid.
**pnEK-vH-PieF_1-109**		1-109	His_6 (N) / TEV	Amplified from wild-type plasmid. Used as a template for generation of PieF 2M mutant (E117R; L121E)
**pnEK-vH-PieF_5M**	His_6-PieF 5M	1-125	His_6 (N) / TEV	Amplified from synthetic PieF 5M gene (provided by GenScript) and inserted into pnEK-vH<br><br>5M = T98E; F111E; R113E; E117R; L121E
**pnEK-vH-PieF_2M**	His_6-PieF 2M	1-125	His_6 (N) / TEV	Amplified from pnEK-vH-PieF_1-109<br>Mutations were introduced in a reverse primer.<br>2M = E117R; L121E
**pnEK-vH-PieF_3M**	His_6-PieF 3M	1-125	His_6 (N) / TEV	Amplified from synthetic PieF 3M gene (provided by GenScript) and inserted into pnEK-vH<br>3M = T98E; F111E; R113E
**pET28b-SUMO-NOT7**	His_6-SUMO-NOT7<br><br>NOT7 (only in NOT6:NOT7 heterodimer)	1-285	His_6-SUMO (N) / TEV	Used to generate His_6-SUMO-NOT7 and NOT6:NOT7 heterodimer<br><br>For details see Raisch et al, 2019

**pnYC-vS-SUMO-NOT7**	StrepII-SUMO-NOT7 in His_6-NOT6: StrepII-SUMO-NOT7 heterodimer.	1-285	StrepII (N) / TEV	For generation of His_6-NOT6: StrepII-SUMO-NOT7
**pET28b-StrepII-SUMO-NOT7**	His_6-StrepII-SUMO-NOT7	1-285	StrepII (N) / TEV	For the generation of His_6-StrepII-SUMO-NOT7
**pnYC-pM-StrepII-NOT7**	StrepII-NOT7	1-285	MBP (N) / 3Cpro	For generation NOT6Δ_{1-28}:StrepII-NOT7
**pMCSG19c-NOT6**	NOT6 His_6-NOT6 in His_6-NOT6: StrepII-SUMO-NOT7 heterodimer.	1-557	MBP (N)<br><br>His_6 (N) / TEV	Used to generate NOT6:NOT7 and His_6-NOT6: StrepII-SUMO-NOT7<br><br>MBP is proteolytically cleaved in vivo.<br><br>For details see Raisch et al, 2019
**pnEA-pM-NOT6Δ_{1-28}**	NOT6Δ_{1-28}: Strep-NOT7	29-557	MBP (N) / 3Cpro	For generation of NOT6Δ_{1-28}:StrepII-NOT7
**pnEK-NSupH-NOT8**	His_6-SUMO-NOT8	1-292	His_6-SUMO (N) / 3Cpro	For all experiments.
**pnYC-pH-NOT1_1093-1317**	His_6-NOT1 (1093-1317)	1093-1317	His_6 (N) / 3Cpro	For details see Petit et al, 2012
**pnYC-pM-NOT1_1351-1588**	NOT1 (1351-1588)	1351-1588	MBP (N) / 3Cpro	For details see Chen et al, 2014<br><br>Used for the production of NOT9 module
**pnEA-NOT9_19-285**	NOT9 (19-285)	19-285	-	For details see Chen et al, 2014
**pnEK-NSupH-NOT1_1093-1317-Strep**	His_6-SUMO-NOT1 (1093-1317)-StrepII	1093-1317	His_6-SUMO (N) / 3Cpro<br><br>StrepII (C)	StrepII-tag added through incorporation in the reverse primer. pnYC-pH-NOT1_1093-1317 was used as a template
**pnYC-pHM-RNF219-(439-600)-Strep**	His_6-MBP-RNF219 (439-600)-StrepII	439-600	His_6-MBP (N)/3Cpro<br><br>StrepII (C)	Used for pull-down in Fig.1C as a positive control for the NOT9 module<br><br>For details see Poetz et al, 2021

**pFBDM-NOT6L:His$_8$-NOT7**	NOT6L:<br>His8-NOT7	NOT6L<br>1-557<br><br>NOT7<br>1-285	His$_8$ (N)<br>/TEV on NOT7	For the production of NOT6L:His$_8$-NOT7 heterodimer in Sf21 insect cells
**mEGFP-C1_PieF**	GFP-PieF	1-125	GFP (N)	For the production of GFP-PieF in HEK293 cells.<br><br>PieF sequence was amplified from the pnEK-vH-PieF plasmid.<br><br>Served as a template for the generation of the pnEK-vH-GFP-PieF plasmid.
**mEGFP-C1_PieF 5M**	GFP-PieF 5M	1-125	GFP (N)	For the production of GFP-PieF 5M in HEK293 cells.<br><br>PieF 5M sequence was amplified from pnEK-vH-PieF 5M plasmid
**pnEK-vH-GFP-PieF**	His$_6$-GFP-PieF	1-125	His$_6$-GFP (N)	For the production of the His$_6$-GFP-PieF.<br><br>GFP-PieF sequence was amplified from mEGFP-C1_PieF and inserted into pnEK-vH
**mEGFP-C1_NOT7^{D40A}**	GFP-NOT7^{D40A}	1-285	GFP (N)	For the production of GFP-NOT7^{D40A} in HEK293 cells.<br><br>D40A mutation is catalytically inactive.<br><br>NOT7^{D40A} was amplified from pET28b-SUMO-NOT7^{D40A}
**pET33b-PARN**	PARN	1-639	His$_6$ (N) / thrombin	

Supplementary Table 4. Hydrogen bonds and salt bridges in NOT1:NOT7:PieF model (PDBe PISA interface).

	Residue [atom], **NOT7**		Residue [atom], **PieF**
Hydrogen bonds		Distance, [Å]	
1	ASN 173[ND2]	2.88	THR 98[OG1]
2	CYS 67[SG]	3.63	SER 99[OG]
3	ILE 51[N]	3.25	GLY 100[O]
4	CYS 67[SG]	3.70	GLY 100[O]
5	ARG 49[N]	2.98	MET 102[O]
6	ARG 49[NH2]	3.26	GLU 117[OE1]
7	ARG 49[NH1]	3.21	GLU 117[OE2]
8	LEU 209[N]	3.36	ASN 123[O]
9	HIS 225[NE2]	3.00	ASN 123[OD1]
10	HIS 157[ND1]	3.73	LYS 124[O]
11	ASP 106[O]	2.76	LYS 87[NZ]
12	ARG 49[O]	2.50	MET 102[N]
13	ASP 111[OD2]	3.15	GLU 114[N]
14	PHE 156[O]	2.51	LYS 124[NZ]
15	ASP 161[OD2]	2.93	LYS 124[NZ]
Salt bridges			
1	ARG 49[NH1]	3.68	GLU 117[OE1]
2	ARG 49[NH2]	3.26	GLU 117[OE1]
3	ARG 49[NH1]	3.21	GLU 117[OE2]
4	ARG 49[NH2]	3.88	GLU 117[OE2]
5	ASP 161[OD2]	2.93	LYS 124[NZ]
6	ASP 40[OD1]	3.33	LYS 124[NZ]
	Residue [atom], **NOT7**		Residue [atom], **NOT1**
Hydrogen bonds		Distance, [Å]	
1	ARG 32[NH1]	3.63	THR1213[OG1]
2	ASN 171[ND2]	3.64	PRO1255[O]
3	THR 142[O]	2.97	HIS1212[N]
4	GLU 138[OE2]	3.38	LYS1218[NZ]
Salt bridges		Distance, [Å]	
1	ARG 32[NH1]	3.63	THR1213[OG1]

Supplementary Table 5. Hydrogen bonds and salt bridges in NOT1:NOT8:PieF model (PDBe PISA interface).

	Residue [atom], **NOT8**		Residue [atom], **PieF**
Hydrogen bonds		Distance, [Å]	
1	ASP 106[O]	3.68	LYS 87[NZ]
2	ARG 49[O]	2.96	MET 102[N]
3	ASP 111[OD2]	2.49	GLU 114[N]
4	ASP 111[OD2]	3.05	PRO 115[N]
5	ASP 161[OD2]	2.90	LYS 124[NZ]
6	ASP 40[OD1]	3.54	LYS 124[NZ]
7	ARG 173[NH2]	3.31	LYS 96[O]
8	ARG 173[NH2]	2.59	THR 98[OG1]
9	ARG 49[N]	2.98	MET 102[O]
10	ARG 49[NH2]	3.72	GLU 114[OE1]
11	ARG 49[NH2]	2.83	GLU 117[OE2]
12	LEU 209[N]	3.58	ASN 123[O]
Salt bridges			
1	ASP 161[OD2]	2.90	LYS 124[NZ]
2	ASP 40[OD1]	3.54	LYS 124[NZ]
3	ARG 49[NH2]	3.72	GLU 114[OE1]
4	ARG 49[NH2]	3.32	GLU 117[OE1]
5	ARG 49[NH1]	3.07	GLU 117[OE2]
6	ARG 49[NH2]	2.83	GLU 117[OE2]
	Residue [atom], **NOT8**		Residue [atom], **NOT1**
Hydrogen bonds		Distance, [Å]	
1	B:HIS1212[N]	3.56	C:THR 142[O]
2	B:ASN1256[ND2]	3.22	C:MET 141[SD]

Supplementary Table 6. Protein sequence database codes used for bioinformatic analyses in Supplementary Figures 5, 8-11.

Protein	NCBI accession code
Legionella pneumophila PieF	WP_010947688.1
Legionella sp. km772 PieF	WP_127098296.1
Legionella shakespearei PieF	KTD57792.1
Legionella septentrionalis PieF	WP_127111431.1
Legionella waltersii PieF	WP_058480986.1
Legionella massiliensis PieF	WP_043872940.1
Legionella tunisiensis PieF	WP_156812310.1
Legionella feelii PieF	WP_131753139.1
Legionella 31fl33 PieF	WP_228363078.1
Legionella donaldsonii PieF	WP_147285514.1
Homo sapiens NOT7	NP_001309019.1
Homo sapiens NOT8	NP_001288002.1
Acanthamoeba castellanii NOT7	XP_004337732.1
Dictyostelium discoideum NOT7	XP_638651.1
Tetrahymena thermophila NOT7	EAS03892.2
Naegleria gruberi NOT7	XP_002679115.1
Cercozoa sp NOT7	MES1909362.1

Supplementary Figure 1

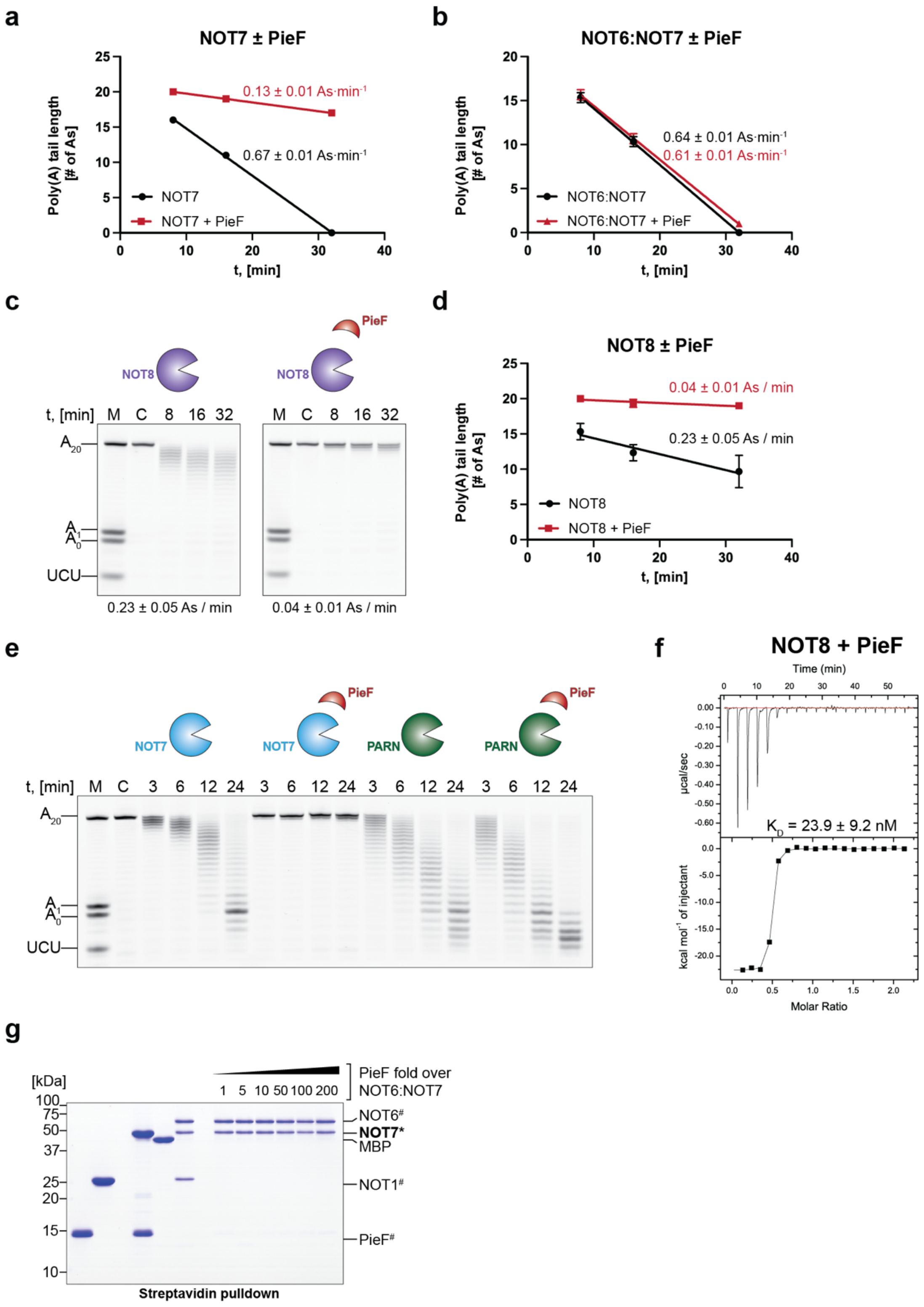

Supplementary Fig. 1. Characterization of NOT7 and NOT8 deadenylases as main PieF targets.

(a-b) Quantitation of deadenylation assays with His_6-SUMO-NOT7 (a) and NOT6:NOT7 (b) in the presence and absence of His_6-tagged PieF from Fig. 1d,e. Source data are provided on page 35.

(c) In vitro deadenylation assays with 50 nM of UCUACAU-A_{20} RNA substrate, 500 nM of His_6-SUMO-NOT8 without (left panel) and with (right panel) 500 nM of His_6-tagged PieF. Poly(A) tail length changes were quantified by plotting the most abundant tail length at each time point. Linear regression was used to determine the apparent deadenylation rate (As / min); values are presented as mean ± SE (n = 3). Source data are provided on page 36.

(d) Quantitation of deadenylation assays with His_6-SUMO-NOT8 in the presence and absence of His_6-tagged PieF from Supplementary Fig. 1c. Source data are provided on page 37.

(e) In vitro deadenylation assays with 50 nM of UCUACAU-A_{20} RNA substrate, 500 nM His_6-SUMO-NOT7, and 2 nM PARN without (left panel) and with (right panel) equimolar amount of His_6-tagged PieF to NOT7 and 20-fold excess over PARN. Source data are provided on page 38.

(f) Representative isothermal titration calorimetry (ITC) thermograms of the interaction between His_6-tagged PieF and His_6-SUMO-NOT8. The upper panels show raw data in (µcal sec^{-1}), and the lower panels represent the integration of heat changes associated with each injection (kcal mol^{-1} of injectant). Data were fitted using a one-site binding model. The parameters of the runs are summarized in Supplementary Table 1.

(g) Coomassie-stained 15% polyacrylamide gel of in vitro pull-down assay with immobilized His_6-NOT6:StrepII-NOT7 and titrated 1 to 200-fold excess of PieF over the heterodimer. MBP-StrepII was a negative control, and NOT1-MIF4G (E1093–S1317) was a positive control for His_6-NOT6:StrepII-SUMO-NOT7. # indicates the presence of N-terminal His6 on NOT6, NOT1 and PieF. * indicates a StrepII-SUMO-tag on the N-terminus of NOT7. Source data are provided on page 39.

The schematics in panels c and e were drawn using Adobe Illustrator 2025.

Supplementary Figure 2

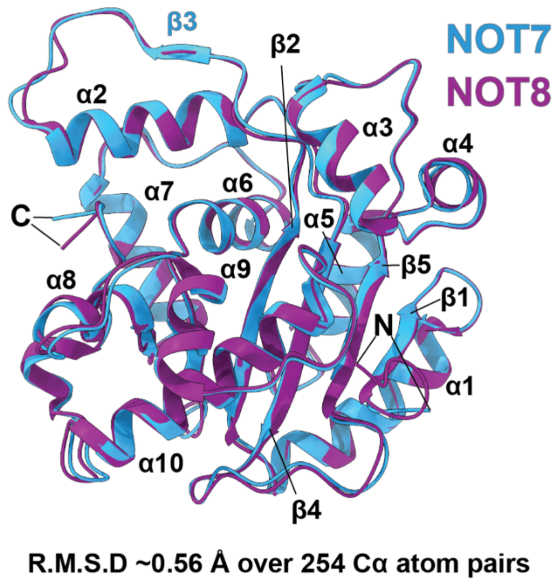

Supplementary Fig.2. Structural comparison of NOT7 and NOT8 deadenylases.

Superposition of NOT8 (purple) and NOT7 (blue) within the NOT1:NOT7/8:PieF.

Supplementary Figure 3

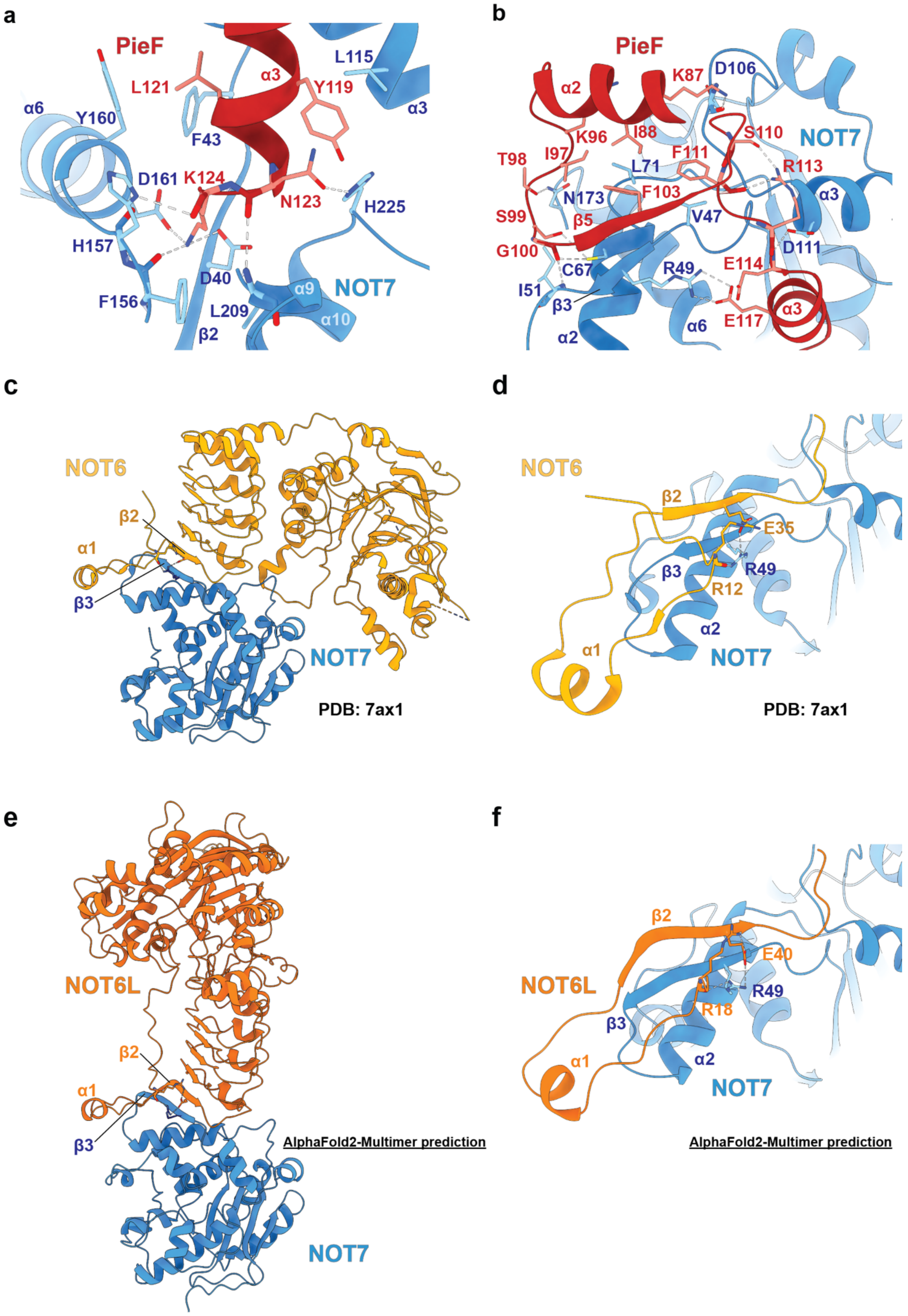

Supplementary Fig. 3. Close-up views of the binding interfaces of NOT7.

(a) Interaction of the C-terminal half of the helix α3 of PieF (red) with the catalytic site of the NOT7 (blue) and adjacent residues. Both proteins are represented as cartoons and individual residues as sticks. Q10–Y34, Y62–M107, S119–S155, L174–G208, and F246–G263 of NOT7 were omitted for clarity. Dashed lines indicate hydrogen bonding. D40 and D161 of NOT7 are additionally involved in salt bridge interactions with $K124^{PieF}$.

(b) Interaction of the PieF (red) with the non-catalytic part of NOT7 (blue). M1–D84 of PieF and Q10–Y34, L174–G222, and F246–G263 of NOT7 were omitted for clarity. Represented as in a. Dashed lines indicate interaction via hydrogen bonding and a salt bridge between $R49^{NOT7}$ and $E117^{PieF}$. The distances between the residues involved in hydrogen bonding and salt bridge interactions between NOT7 and PieF are summarized in the Supplementary Table 4.

(c) Cartoon representation of the NOT6:NOT7 heterodimer from the crystal structure (PDB: 7ax1). β2 strand of NOT6 (yellow) and β3 strand of NOT7 (blue) assemble into a β-sheet stabilized by residue-specific contacts, which include a salt bridge between $R49^{NOT7}$ and $E35^{NOT6}$ and the carbonyl group of $R12^{NOT6}$.

(d) Close-up view of c.

(e) Cartoon representation of the NOT6L:NOT7 heterodimer predicted by the AlphaFold2. β2 strand of NOT6L (orange) and β3 strand of NOT7 (blue) create a β-sheet which is additionally stabilized by residue-specific contacts, which include a salt bridge between $R49^{NOT7}$ and $E40^{NOT6L}$ and the carbonyl group of $R18^{NOT6L}$.

(f) Close-up view of e.

Supplementary Figure 4

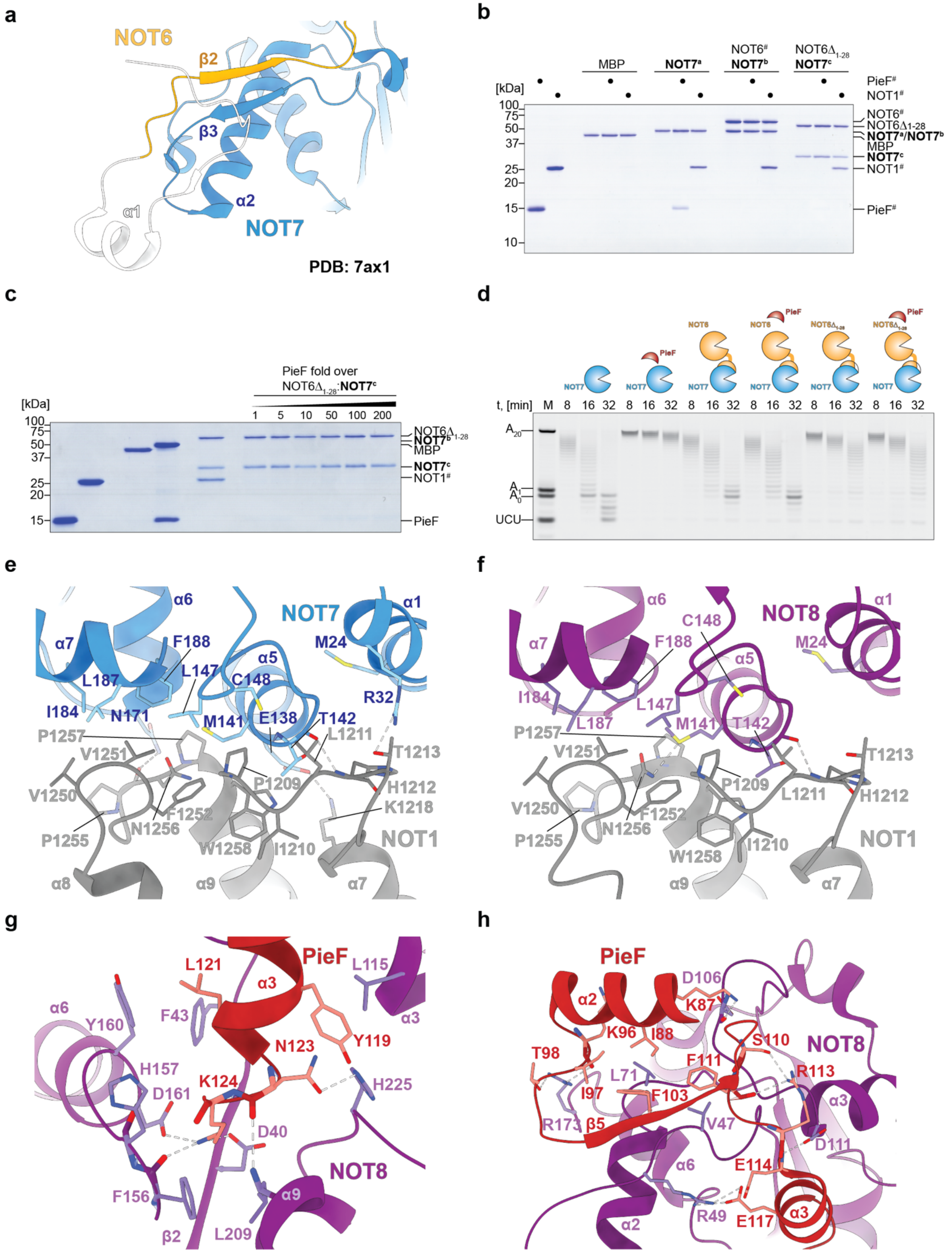

Supplementary Fig. 4 Structure of the NOT1:NOT8:PieF complex.

(a) Cartoon representation of the NOT6:NOT7 heterodimer from the crystal structure (PDB: 7ax1). β2 strand of NOT6 (yellow) and β3 strand of NOT7 (blue) assemble into a β-sheet. The truncated part of NOT6 (up to K28) used in pull-down assays (panels b and c) is colored in white.

(b) Coomassie-stained 15% polyacrylamide gel of in vitro pull-down assay with immobilized NOT6$\Delta_{1\text{-}28}$:StrepII-NOT7. MBP-StrepII was a negative control, and NOT1-MIF4G (E1093–S1317) was a positive control. PieF was added in 100-fold excess. # indicates the presence of N-terminal His_6-tag on NOT6, NOT1 and PieF. "a" indicates His_6-Strep-SUMO-tag on the N-terminus of NOT7; "b" indicates StrepII-SUMO-tag on the N-terminus of NOT7; "c" indicates a StrepII-tag on the N-terminus of NOT7. Source data are provided on page 40.

(c) Coomassie-stained 15% polyacrylamide gel of in vitro pull-down assay with immobilized NOT6$\Delta_{1\text{-}28}$:StrepII-NOT7 and titrated 1 to 200-fold excess of PieF over the heterodimer. MBP-StrepII was a negative control, and NOT1-MIF4G (E1093–S1317) was a positive control for NOT6$\Delta_{1\text{-}28}$:StrepII-NOT7. # indicates the presence of N-terminal His_6-tag on NOT6 and NOT1. "b" indicates a StrepII-SUMO-tag on the N-terminus of NOT7; "c" indicates a StrepII-tag on the N-terminus of NOT7 as in the panel b. Source data are provided on page 41.

(d) In vitro deadenylation assays with 50 nM of UCUACAU-A_{20} RNA substrate, 500 nM of His_6-SUMO-NOT7, 250 nM NOT6:NOT7 and 250 nM NOT6$\Delta_{1\text{-}28}$:StrepII-NOT7 without and with equimolar amount of PieF. The schematics were drawn using Adobe Illustrator 2025. Source data are provided on page 42.

(e) Binding interface between NOT7 (blue) and NOT1 (grey) in a NOT1:NOT7:PieF structure. Both proteins are represented as cartoons and individual residues as sticks. Dashed lines indicate interaction via hydrogen bonding and a salt bridge between $K1218^{NOT1}$ and $E138^{NOT7}$. The distances between the residues involved in hydrogen bonding and salt bridge interactions between NOT7 and PieF are summarized in the Supplementary Table 4.

(f) Binding interface between NOT8 (purple) and NOT1 (grey) in a NOT1:NOT8:PieF structure. Both proteins are represented as cartoons and individual residues as sticks. Dashed lines indicate interaction via hydrogen bonding. The distances between the residues involved in hydrogen bonding between NOT8 and PieF are summarized in the Supplementary Table 5.

(g) Interaction of the C-terminal half of the helix α3 of PieF (red) with the catalytic site of the NOT8 (purple) and adjacent residues. Both proteins are represented as cartoons and individual residues as sticks. V6–Y34, Y62–I113, S119–S155, L174–G208, and F246–G263 of NOT8

were omitted for clarity. Dashed lines indicate hydrogen bonding. D40 and D161 of NOT8 are additionally involved in salt bridge interactions with $K124^{PieF}$.

(h) Interaction of the PieF (red) with the non-catalytic part of NOT8 (purple). M1–D84 of PieF and V6–Y34, L174–G222, and F246–G263 were omitted for clarity. Represented as in g. Dashed lines indicate interaction via hydrogen bonding and a salt bridge between $R49^{NOT8}$ and $E117^{PieF}$. The distances between the residues involved in hydrogen bonding and salt bridge interactions between NOT8 and PieF are summarized in the Supplementary Table 5.

Supplementary Figure 5

```
H.sapiens (NOT7)     -------------MPAATVDHSQRICEVWACNLDEEMKKIRQVIRKYNYVAMDTEFPGVV
H.sapiens (NOT8)     -------------MPAALVENSQVICEVWASNLEEEMRKIREIVLSYSYIAMDTEFPGVV
T.thermophila        MNNQPPTTSKQSLLGDKSSKEGEIIREVWQDNLEKEFLLIQDLAEECQFVALDTEFPGVL
Cercozoa_sp_MHM6MM   ------------------------MVDVWAGNARATMLEIASFLDDYPCVAMDTEFPGVV
A.castellanii        -------------MGSHSNGLEPEIREVWAENLEEEMAHIRDIVEDYPYIAMDTEFPGIV
N.gruberi            MVGYNLVELSDRNTSINDPDSKIKIIDVWKHNLDTEMHKIIHMVEKYPCIAMDTEFPGVV
D.discoideum         ----------------MVTLHTDEIKDVWGYNLDEEMEKIRNLVDDYNYIAMDTEFPGIV

H.sapiens (NOT7)     ARPIGEFRSNADYQYQLLRCNVDLLKIIQLGLTFMNEQGEYPP-GTSTWQFNFKFNLTED
H.sapiens (NOT8)     VRPIGEFRSSIDYQYQLLRCNVDLLKIIQLGLTFTNEKGEYPS-GINTWQFNFKFNLTED
T.thermophila        YQTA-------QTEYLKIKQNADNLNTIQIGLTFAKSDGTYPS--ACTFQFNFAFNKDKD
Cercozoa_sp_MHM6MM   LRTVPRQFASHEHAYDSVKLNVDSLSLIQVGLTFFDRQGRPRP-GRCTFQFNLRFDLERE
A.castellanii        ARPIGNFKSPSEYHYQTLRCNVDLLKIIQLGLTFTDGEGRLPP-GVATWQFNFKFSLTED
N.gruberi            ARPVGNFRTQSEYHYQTLKCNVNLLRVIQLGLTFTDLQGNVPDDGQCVWQFNFKFNLKED
D.discoideum         TRPVGNFRSTSDYHYQTLRLNVDQLKIIQLGLTFSDSEGNLAK-PTCTWQFNFKFSLSED

H.sapiens (NOT7)     MYAQDSIELLTTSGIQFKKHEEEGIETQYFAELLMTSGVVLCEGVKWLSFHSGYDFGYLI
H.sapiens (NOT8)     MYSQDSIDLLANSGLQFQKHEEEGIDTLHFAELLMTSGVVLCDNVKWLSFHSGYDFGYMV
T.thermophila        SCNKEAIKFLEESGIQFKDHARRGIQPADFAELMYSSGLLFNEDICWVTFHGGFDYCYFL
Cercozoa_sp_MHM6MM   LFAPDSIALLREAGIDFQRCAREGIPAEELAELFLTSGLVLNHRVHWIVFHGAYDQAYLL
A.castellanii        MYAQDSIDLLTRSGINFKRHEEHGVDVSHFGELLTSSGIVLDDRIKWISFHSGYDFGYLL
N.gruberi            MYAQDSIELLADSGIKFLSHEKEGIDVETFGEKLISSGIVLSEDVKWISFHSAYDFGYLI
D.discoideum         MYAQDSIDLLSRSGIEFKKNEANGIDILDFGEQLMSSGIVLNDNIKWISFHSGYDFGYLL

H.sapiens (NOT7)     KILTNSNLPEEELDFFEILRLFFPVIYDVKYLMKSCKNLK-GGLQEVAEQLELERIGPQH
H.sapiens (NOT8)     KLLTDSRLPEEEHEFFHILNLFFPSIYDVKYLMKSCKNLK-GGLQEVADQLDLQRIGRQH
T.thermophila        KTLIDQKLPNTCKEFYEQQHHYFPLSIDVKLIIQEIDGFKYLGLEKLSKSLDLERIGPQH
Cercozoa_sp_MHM6MM   KLLSAQNLPDQLSEFRESCLLYFPHTSDLKCLTS--GLIH-GGLDAVAQQLRVKRTGSAH
A.castellanii        KILTCKPLPAQEEEFFELLLAYFPCIYDIKYLMKSCKSLK-GGLNELANDLEVERIGPQH
N.gruberi            KLLTNDPLPEVERDFFELLHTFFPCIYDIKYLMRSCDTLK-GGLNQLAEDLGLRRIGPAH
D.discoideum         KSLTCTVLPLDEADFFGSARTYFPCIYDIKYIMKSCKNLK-GGLSELADDLDIKRIGPQH

H.sapiens (NOT7)     QAGSDSLLTGMAFFKMREMFFEDHID-------------DAKYCGHLYGLGSGSSYVQ--
H.sapiens (NOT8)     QAGSDSLLTGMAFFRMKELFFEDSID-------------DAKYCGRLYGLGTGVAQKQ--
T.thermophila        QAGSDSLMTMKVYFKLKEREKIN----------------FDSIKNQIFGLSEYEDERQLN
Cercozoa_sp_MHM6MM   QAGSDSRVTGDAFFALERACNDESEASSRPPWYTHVRQRRDLVCNSIYGLRSENTRPELQ
A.castellanii        QAGSDSLLTSATFFKMKRMFFENNLD-------------DSKFLGVLYGLGHPYKAGGYE
N.gruberi            QAGSDSLLTSATFFKMMKVFFENNMN-------------DKKYIGILYGLGEGFDQNHGL
D.discoideum         QAGSDSLLTSTTFFKMRKMFFENQLD-------------DSKYLNILYGLSSFGPDGTPT

H.sapiens (NOT7)     ---NGTGNAYEEEANKQS------------------------------------------
H.sapiens (NOT8)     ---NEDVDSAQEKMSILAI---------------------INNMQQ--------------
T.thermophila        SQS----QQYYEQQQYQSIFNQDYMAGQYYYQNLQGPVMNELFANEYSQMG---VQGSMF
Cercozoa_sp_MHM6MM   QT----------------------------------------------------------
A.castellanii        QQV---------------------------------------------------------
N.gruberi            GYKNGNKKIGNVQ--N------------IHMQSISDS--NAEIATDYT-----------
D.discoideum         NIHTGAGNNPPPQLSNSGSTN-------YGYSPLSQQN-NPNNITNYNSTNPASQNQPTY

H.sapiens (NOT7)     ----------------------------------------------
H.sapiens (NOT8)     ----------------------------------------------
T.thermophila        VRNYSNNT-------FPN---NEQRGGQPRRTPSQR----------
Cercozoa_sp_MHM6MM   ----------------------------------------------
A.castellanii        ----------------------------------------------
N.gruberi            ----------------------------------------------
D.discoideum         YNNYPTTPTRYHNSSGPYSPTGNNLSMSQPNTPSKNNSNDYYHKKS
```

Supplementary Fig. 5 Multiple sequence alignment of NOT7 homologs of *Legionella* hosts. Residues of NOT7/8 from human and protist *Legionella* hosts that are entirely or highly conserved are highlighted in dark blue with white font and dark cyan with black font, respectively. The secondary structure on top of the aligned sequences was assigned based on NOT7 from the NOT1:NOT7:PieF structure. Residues of NOT7 involved in the interaction with PieF are marked with black triangles. Residues of NOT7 engaged in the interaction with NOT6 are marked with orange triangles. Residues of NOT7 involved in the coordination of Mg^{2+} are marked with yellow circles. Orange and pink boxes highlight differences between the C-terminal parts of NOT7 and NOT8, respectively. Alignment was done using the Clustal Omega module of the Geneious Prime, and the output was further processed in ESPript 3.0 (https://endscript.ibcp.fr). The source sequences are listed in Supplementary Table 6.

Supplementary Figure 6

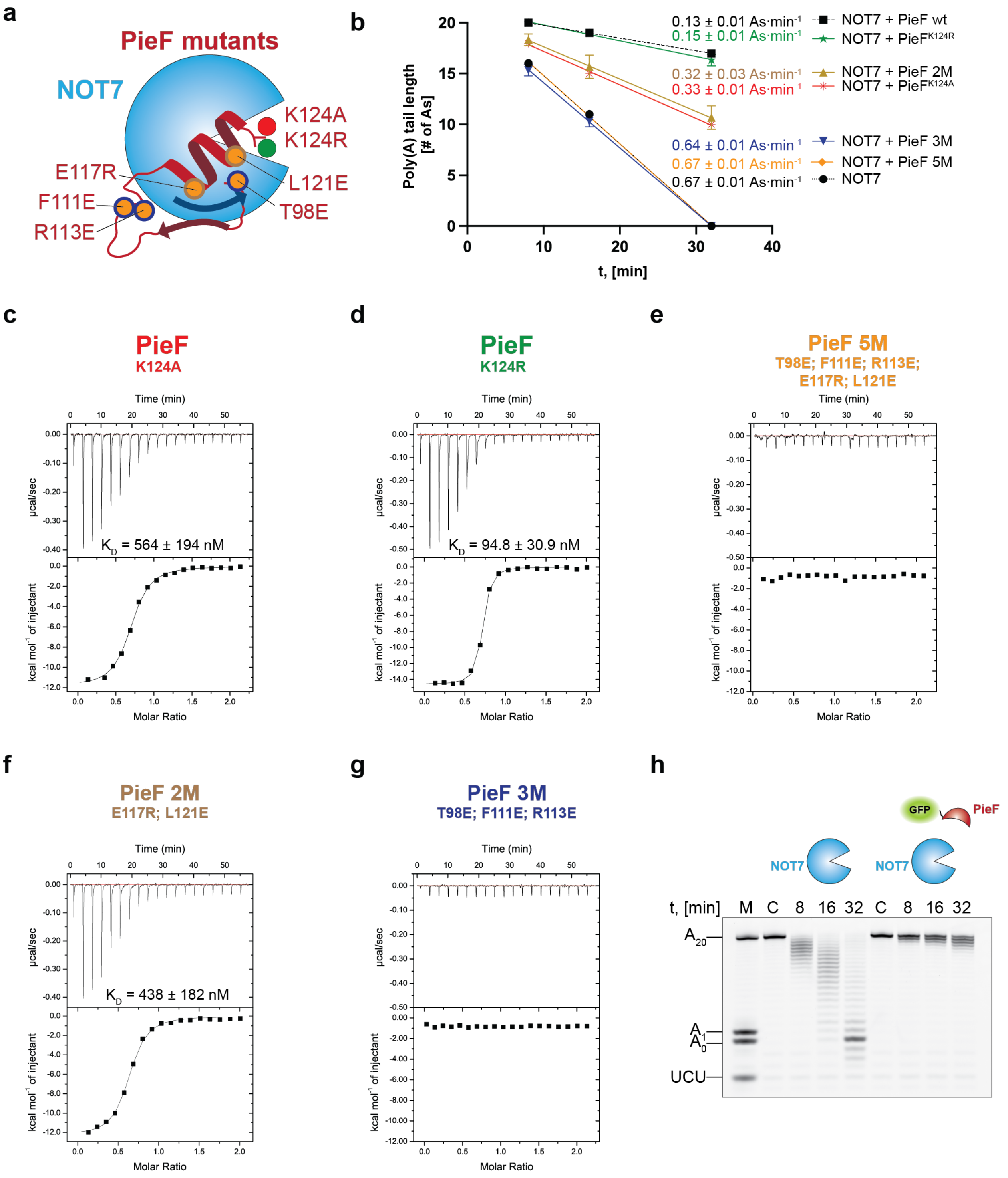

Supplementary Fig. 6. In vitro functional validation of the NOT7:PieF interactions.

(a) Schematic representation of the mutated residues in PieF used in this study (from Fig. 4a).

(b) Quantitation of deadenylation assays with His_6-SUMO-NOT7 in the presence and absence of His_6-tagged PieF K124A, K124R, 5M, 2M, and 3M mutants from Fig. 4b. NOT7 and NOT7 + PieF curves are taken from Supplementary Figure 1 for comparison. Linear regression was used to determine the apparent deadenylation rate (As / min); values are presented as mean ± SE (n = 3). Source data are provided on page 43.

(c-g) Representative ITC thermograms of the interaction between His_6-tagged PieF K124A (c), K124R (d), 5M (e), 2M (f), 3M (g) mutants and His_6-SUMO-NOT7. Binding of PieF 5M and 3M mutants to NOT7 was not detected. The upper panels show raw data in (µcal sec^{-1}), and the lower panels represent the integration of heat changes associated with each injection (kcal mol^{-1} of injectant). Data were fitted using a one-site binding model. The parameters of the runs are summarized in Supplementary Table 1.

(h) In vitro deadenylation assays with 50 nM of UCUACAU-A_{20} RNA substrate, 500 nM of His_6-SUMO-NOT7 (500 nM) without (left panel) and with (right panel) 500 nM of His_6-GFP-PieF. The schematics were drawn using Adobe Illustrator 2025. Source data are provided on page 44.

Supplementary Figure 7

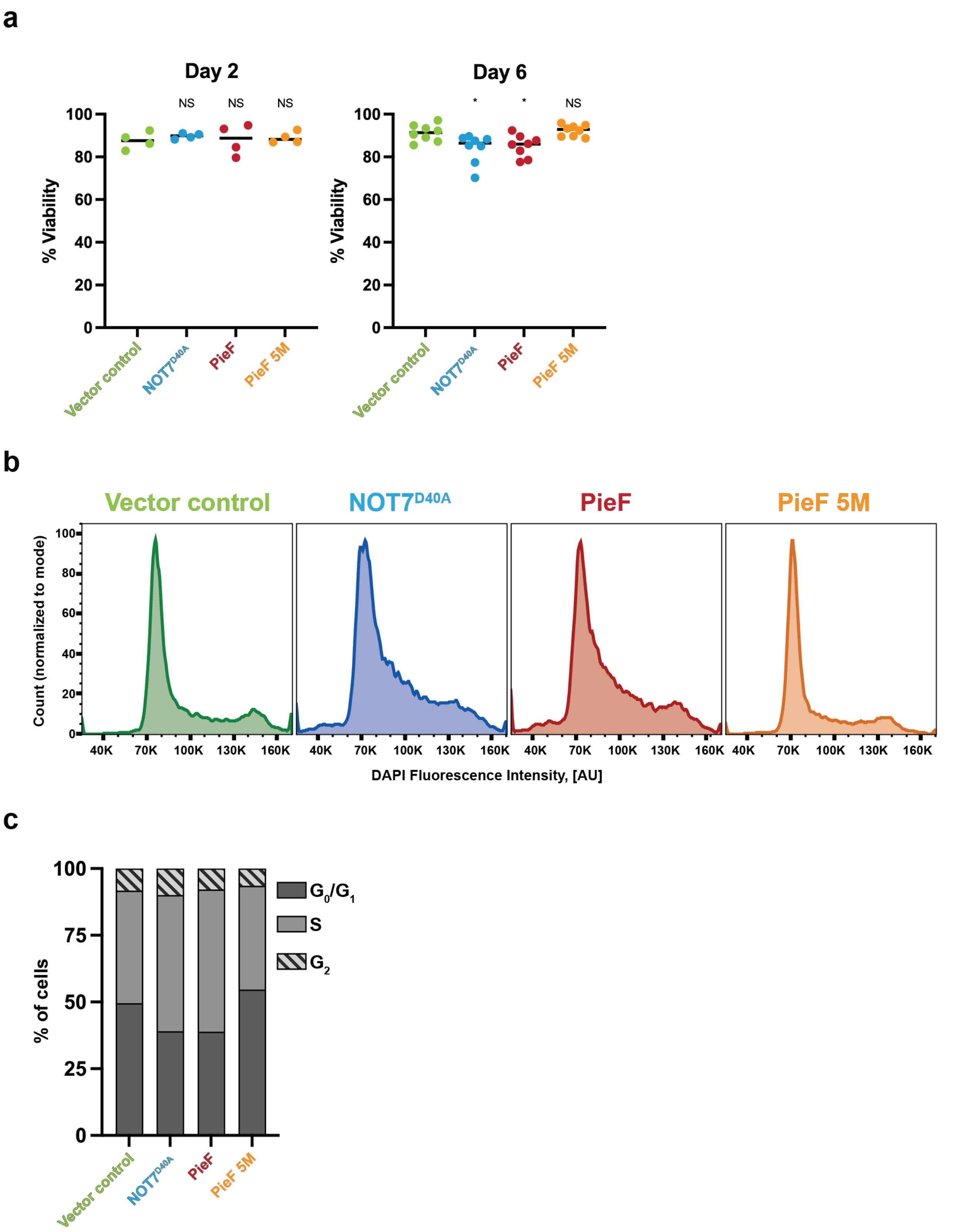

Supplementary Fig. 7. PieF inhibits cell expansion by limiting cell cycle progression and reducing viability.

(a) The percentage of viable cells on day 2 and day 6 after transfection was determined by the exclusion of trypan blue. Asterix indicates statistical significance of $p<0.05$ in two-tailed unpaired students t-test when compared with the control. NS indicates a lack of statistical significance $p\geq0.05$. Source data are provided on page 45.

(b) Flow cytometry analysis of cellular DNA stained with DAPI on day 2 after transfection of GFP control (green), GFP-NOT7^{D40A} (blue), GFP-PieF WT (red) or GFP-PieF 5M (orange). The gating strategy is summarized in the Supplementary Figure 14.

(c) The percentage of cells in each cell cycle phase for the samples from panel B (G_0/G_1, S, G_2). Source data are provided on page 46.

Supplementary Figure 8

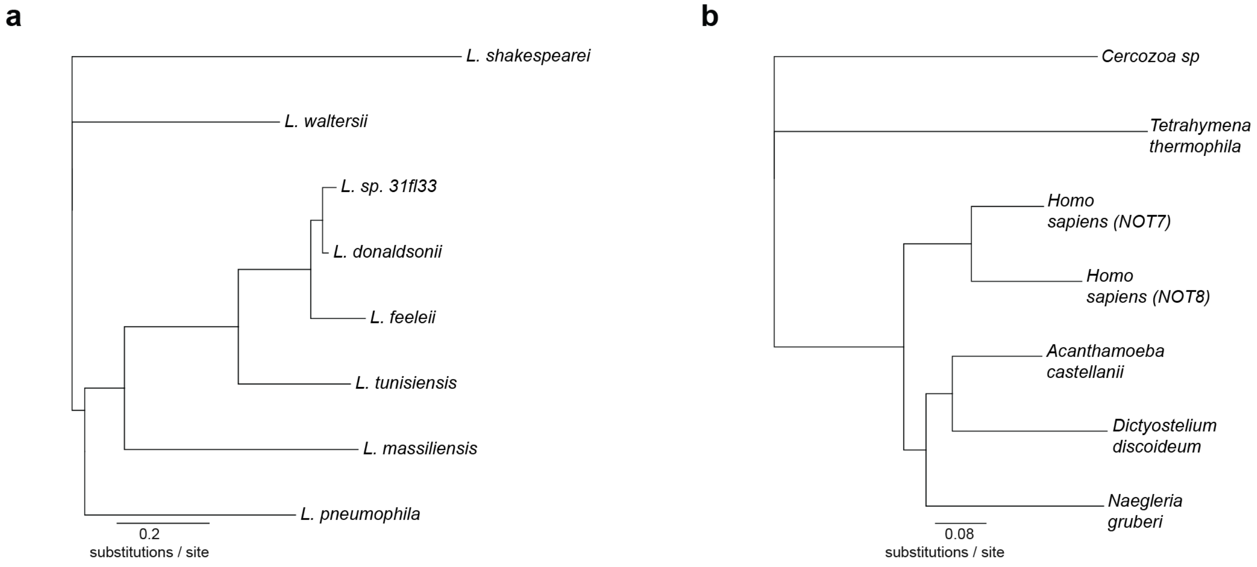

Supplementary Fig. 8. Dendrograms of PieF and NOT7.

(a) A PieF dendrogram of *Legionella* species. A bar indicates the number of substitutions per site. The dendrogram was generated using Geneious Prime suite v2024. The source sequences are listed in Supplementary Table 6.

(b) A NOT7 dendrogram of human and protist hosts of *Legionella pneumophila*. A bar indicates the number of substitutions per site. The dendrogram was generated using Geneious Prime suite v2024. The source sequences are listed in Supplementary Table 6.

Supplementary Figure 9

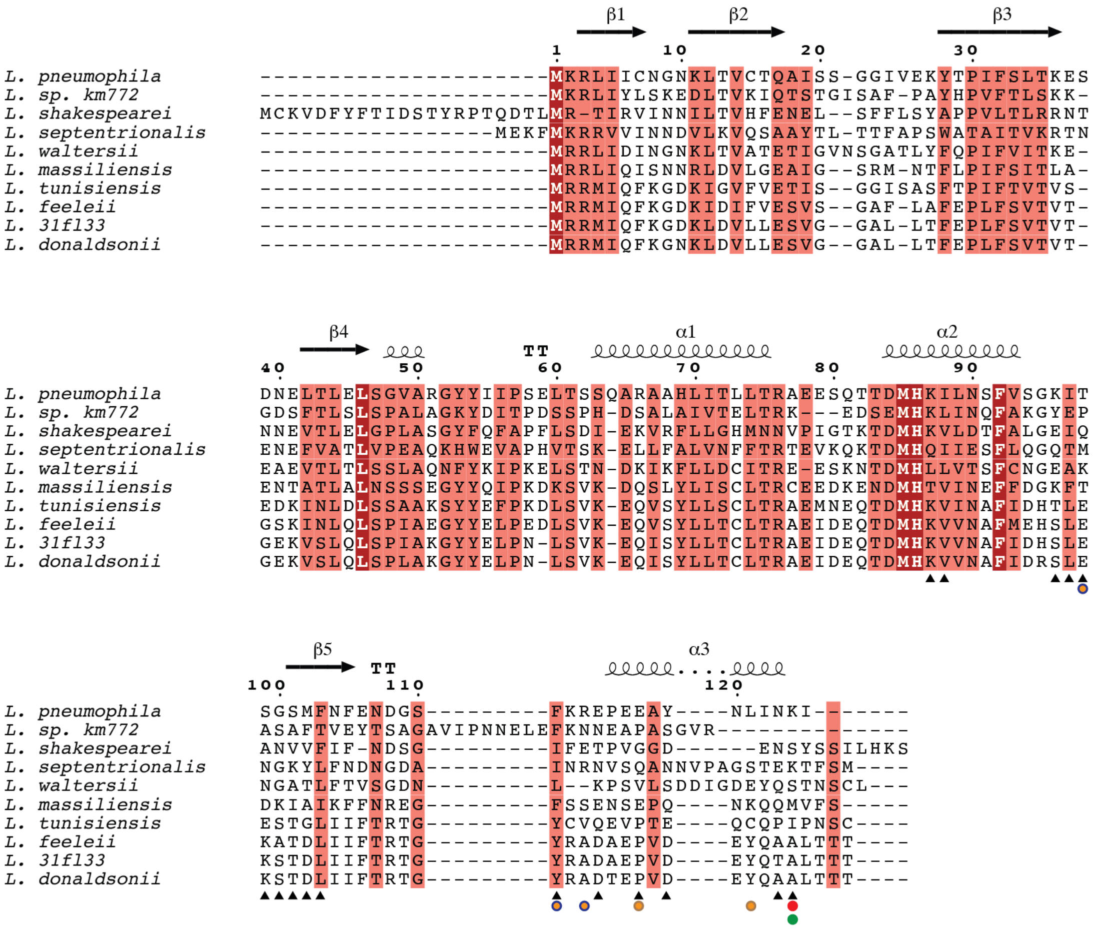

Supplementary Fig. 9. Multiple sequence alignment of PieF homologs from *Legionella* species. Residues of PieF homologs from various *Legionella* species that are invariant or highly conserved are highlighted in dark red with white font and dark pink with black font, respectively. A secondary structure on top of the aligned sequences was assigned based on *L. pneumophila* PieF. Residues involved in the interaction between *L. pneumophila* PieF and NOT7 are marked with black triangles. Residues of *L. pneumophila* PieF mutated in the study are marked in circles. The color scheme of the mutated residues is consistent with Fig. 4a and Supplementary Fig. 6a. Alignment was done with the Clustal Omega module, part of the Geneious Prime suite v2024. The output was further processed in ESPript v3.0 (https://endscript.ibcp.fr). The source sequences are listed in Supplementary Table 6.

Supplementary Figure 10

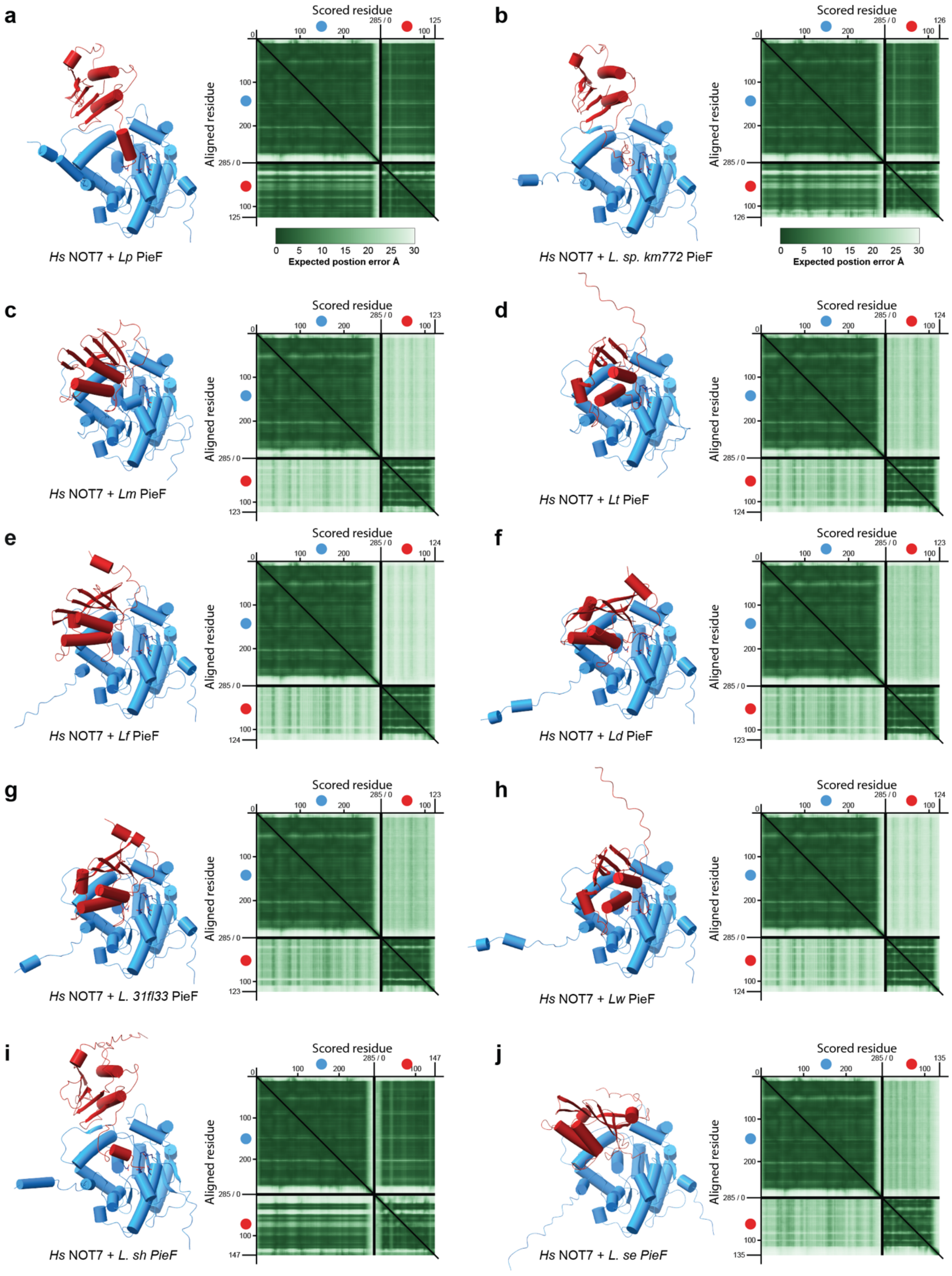

Supplementary Fig. 10. AlphaFold2-Multimer structure predictions of human NOT7 in complex with various *Legionella* PieF homologs.

(a-j) AlphaFold2-Multimer structure prediction of NOT7 (blue) and PieF (red) of *L. pneumophila* (a), *L. sp. km772* (b), *L. massiliensis* (c), *L. tunisiensis* (d), *L. feelii* (e), *L. donaldsonii* (f), *L. 31fl33* (g), *L. waltersii* (h), *L. shakespearei* (i), and *L. septentrionalis* (j). The unrelaxed models were used for comparison. The predicted aligned error (PAE) plots next to the structures were generated using PAE viewer (https://subtiwiki.uni-goettingen.de/v4/paeViewerDemo) and they indicate the confidence of prediction of scored vs aligned residue. Blue and red circles indicate the subunit of the complex that was used for alignment. The source sequences are listed in Supplementary Table 6.

Supplementary Figure 11

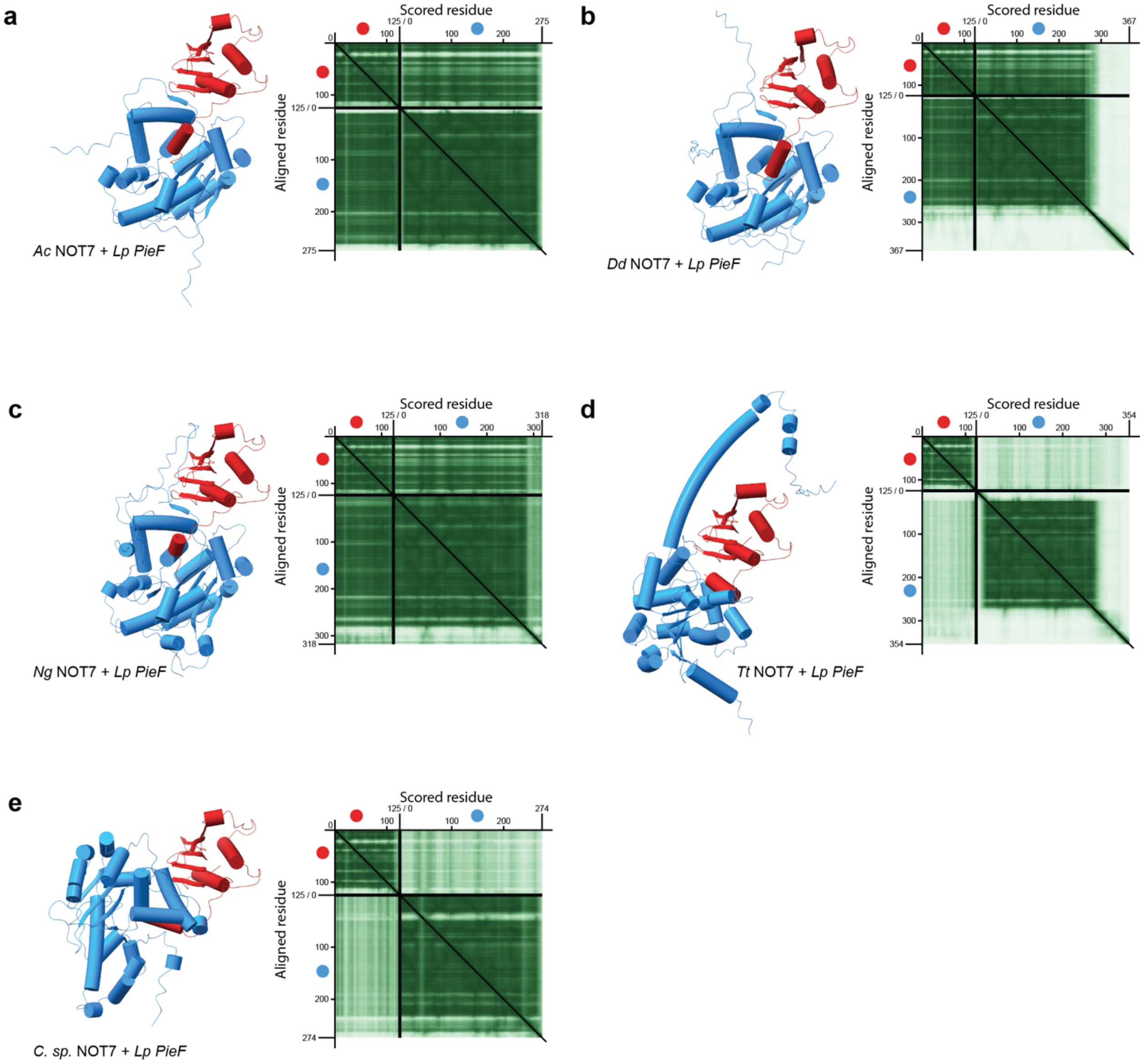

Supplementary Fig. 11. AlphaFold2-Multimer structure predictions of *L. pneumophila* PieF in complex with NOT7 homologs of various hosts.

(a-e) AlphaFold2-Multimer structure prediction of *Lp* PieF (red) and NOT7s (blue) of *A. castellanii* (a), *D. discoideum* (b), *N. gruberi* (c), T. *thermophila* (d), *Cercozoa sp* (e). The unstructured C-terminal region of *D. discoideum* (Y293–S367) is omitted for clarity. The unrelaxed models were used for comparison. The predicted aligned error (PAE) plots next to the structures were generated using PAE viewer (https://subtiwiki.uni-goettingen.de/v4/paeViewerDemo) and they indicate the confidence of prediction of scored vs aligned residue. Blue and red circles indicate the subunit of the complex that was used for alignment. The source sequences are listed in Supplementary Table 6.

Supplementary Figure 12

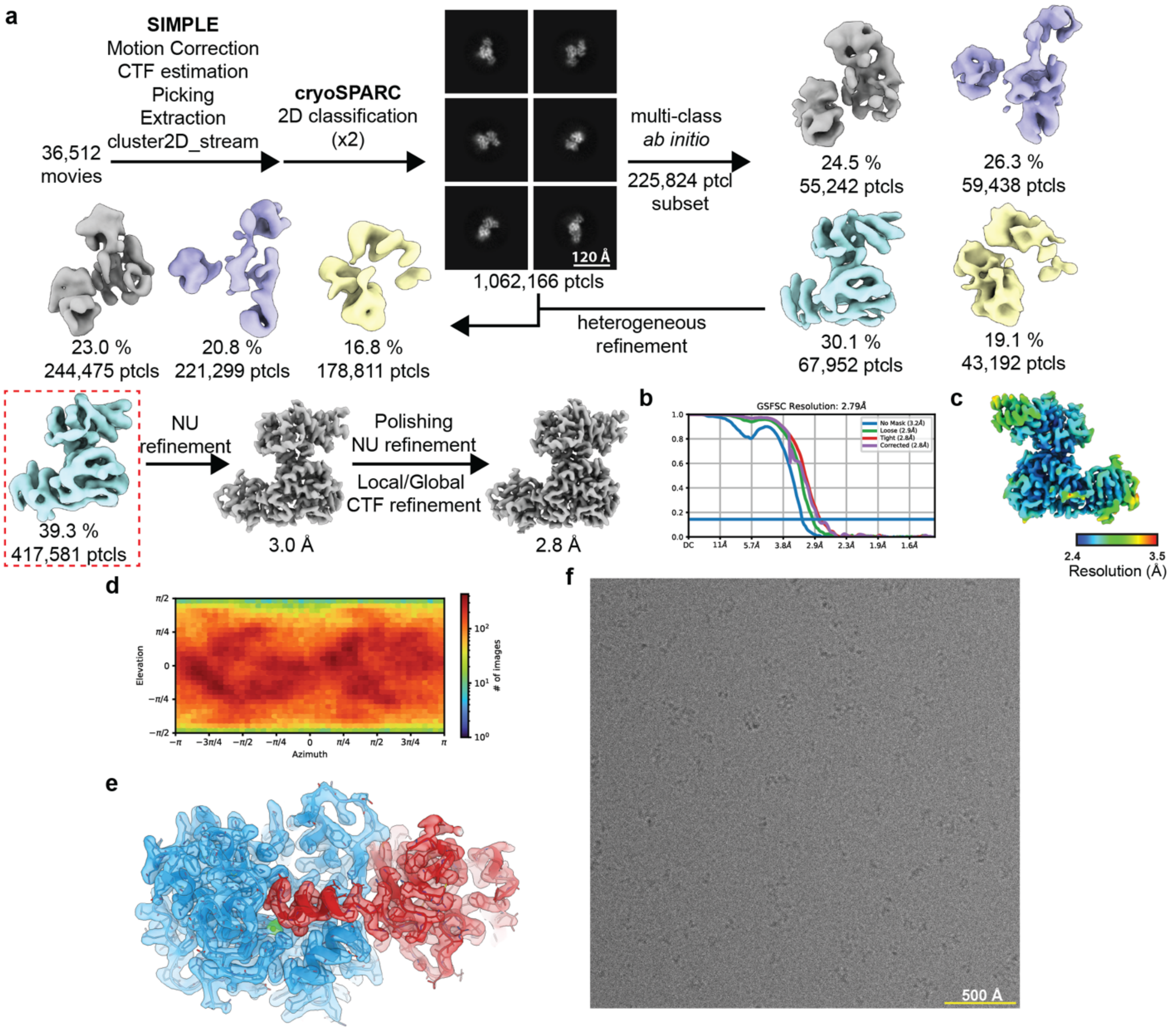

Supplementary Fig. 12. Cryo-EM processing workflow of NOT1:NOT7:PieF with map quality metrics.

(a) Image processing workflow.

(b) Gold-standard Fourier Shell Correlation (FSC) curves used for global resolution estimation.

(c) Local resolution estimate of the volume.

(d) Viewing direction distribution.

(e) Model-to-map overlay for key structural elements. PieF model and density colored red, NOT7 blue.

(f) Representative micrograph.

Supplementary Figure 13

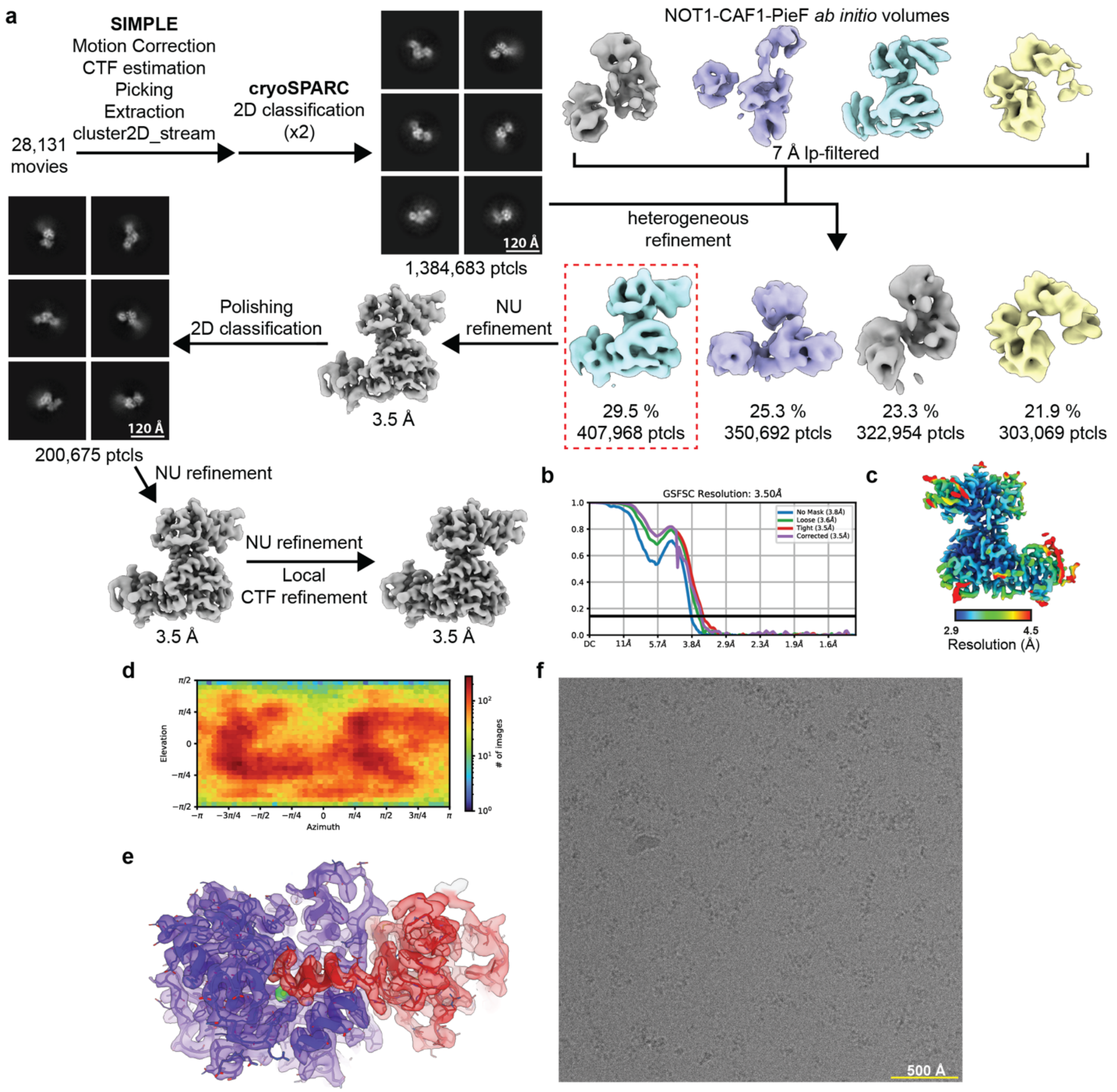

Supplementary Fig. 13. Cryo-EM processing workflow of NOT1:NOT8:PieF with map quality metrics.

- **(a)** Image processing workflow.
- **(b)** Gold-standard Fourier Shell Correlation (FSC) curves used for global resolution estimation.
- **(c)** Local resolution estimate of the volume.
- **(d)** Viewing direction distribution.
- **(e)** Model-to-map overlay for key structural elements. PieF model and density colored red, NOT8 purple.
- **(f)** Representative micrograph.

Supplementary Figure 14

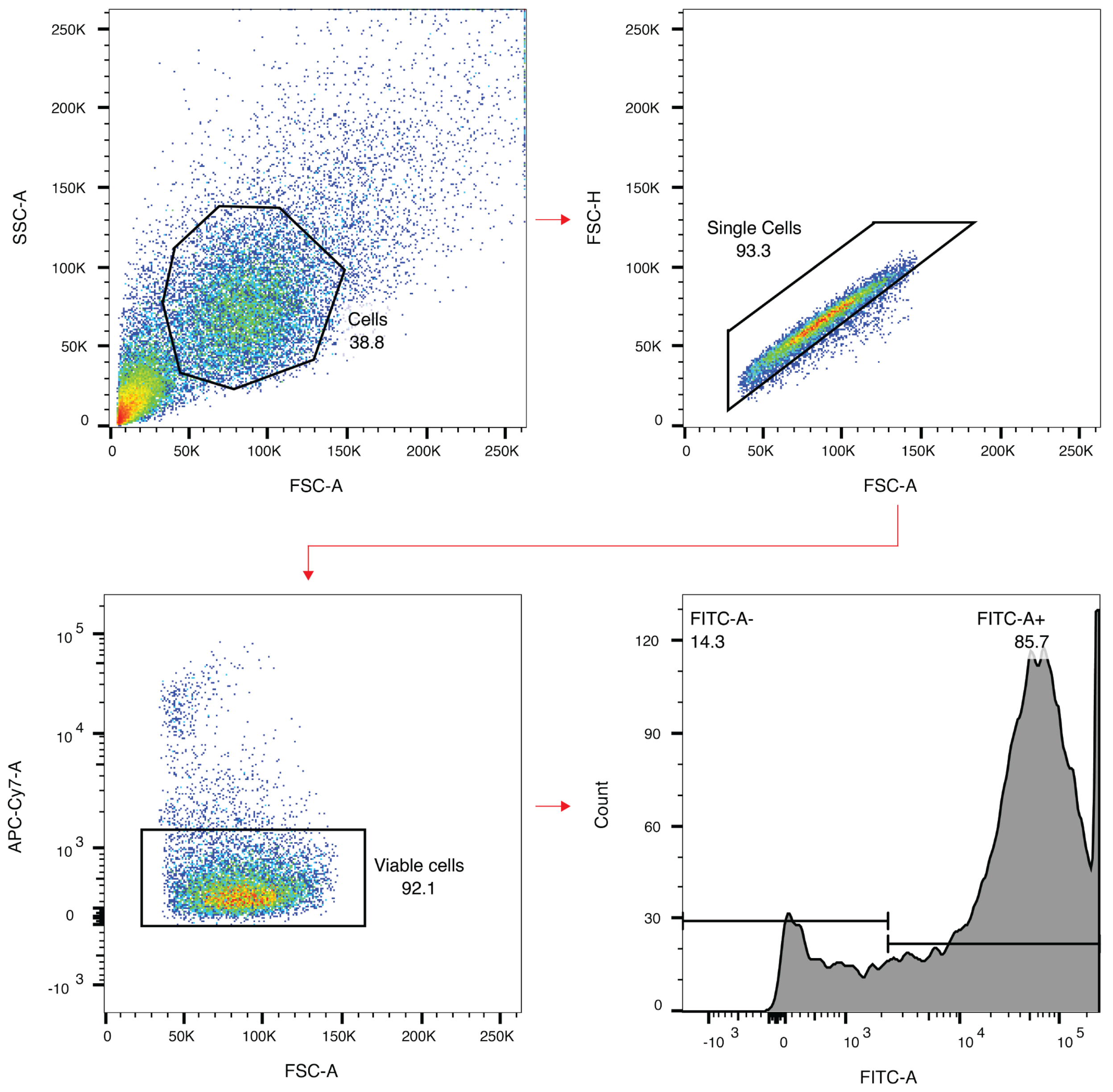

Supplementary Fig. 14. Gating strategy for flow cytometry.

HEK293T cells were gated based on FSC-A and SSC-A, on single cells, on viable cells (ef780 negative), and on GFP positive cells.

Source Data for Supplementary Figure 1a

min	NOT7		
8	16	16	16
16	11	11	11
32	0	0	0

min	NOT7+PieFwt		
8	20	20	20
16	19	19	19
32	17	17	17

Source Data for Supplementary Figure 1b

min	NOT6:NOT7		
8	15	15	16
16	10	10	11
32	0	0	0

min	NOT6:NOT7+PieF		
8	16	15	16
16	11	10	11
32	1	1	1

Source Data for Supplementary Figure 1c

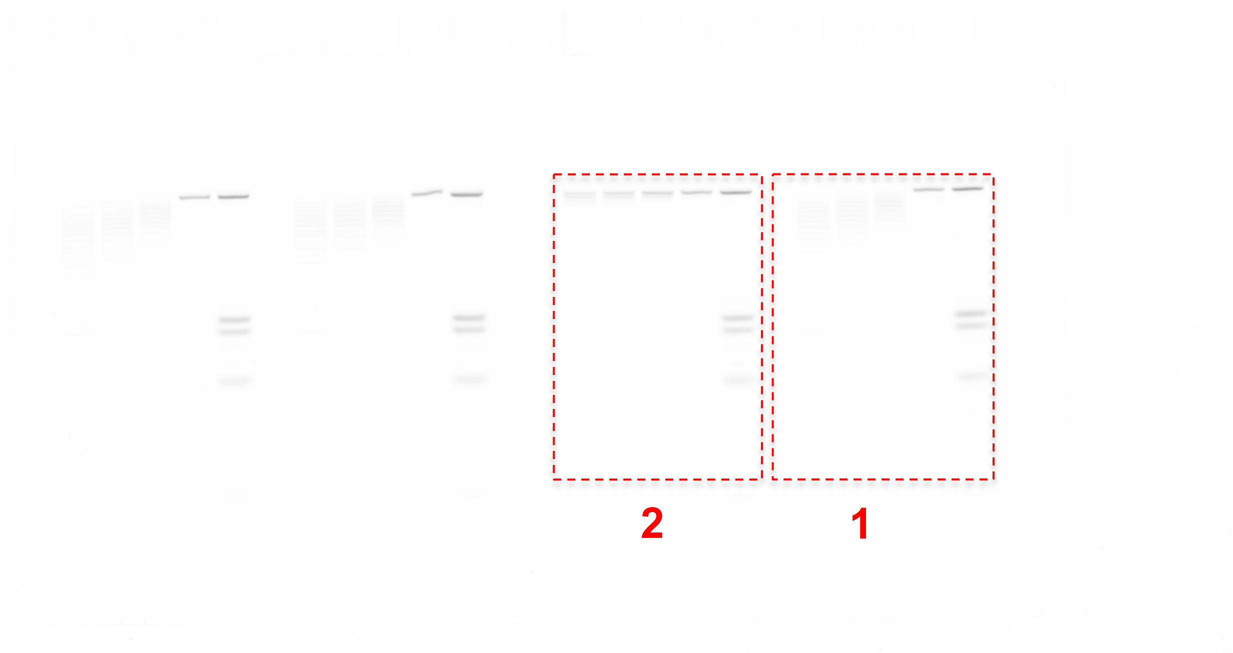

1 denotes left panel in Supplementary Figure 1c (NOT8)

2 denotes right panel in Supplementary Figure 1c (NOT8 + PieF)

Source Data for Supplementary Figure 1d

min	NOT8		
8	16	14	16
16	13	11	13
32	11	7	11

min	NOT8+PieFwt		
8	20	20	20
16	19	19	20
32	19	19	19

Source Data for Supplementary Figure 1e

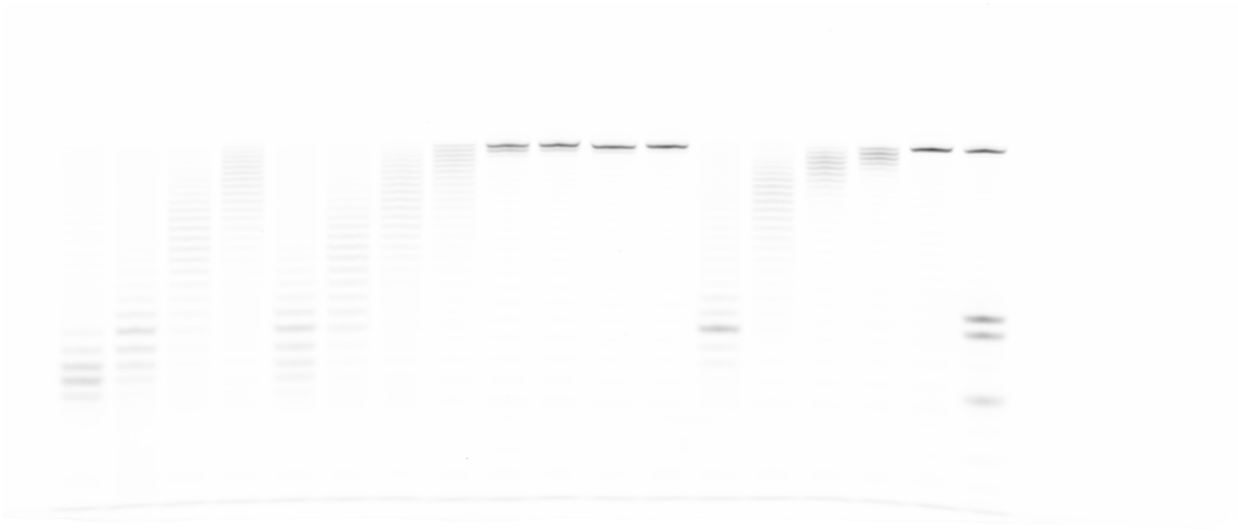

Source Data for Supplementary Figure 1g

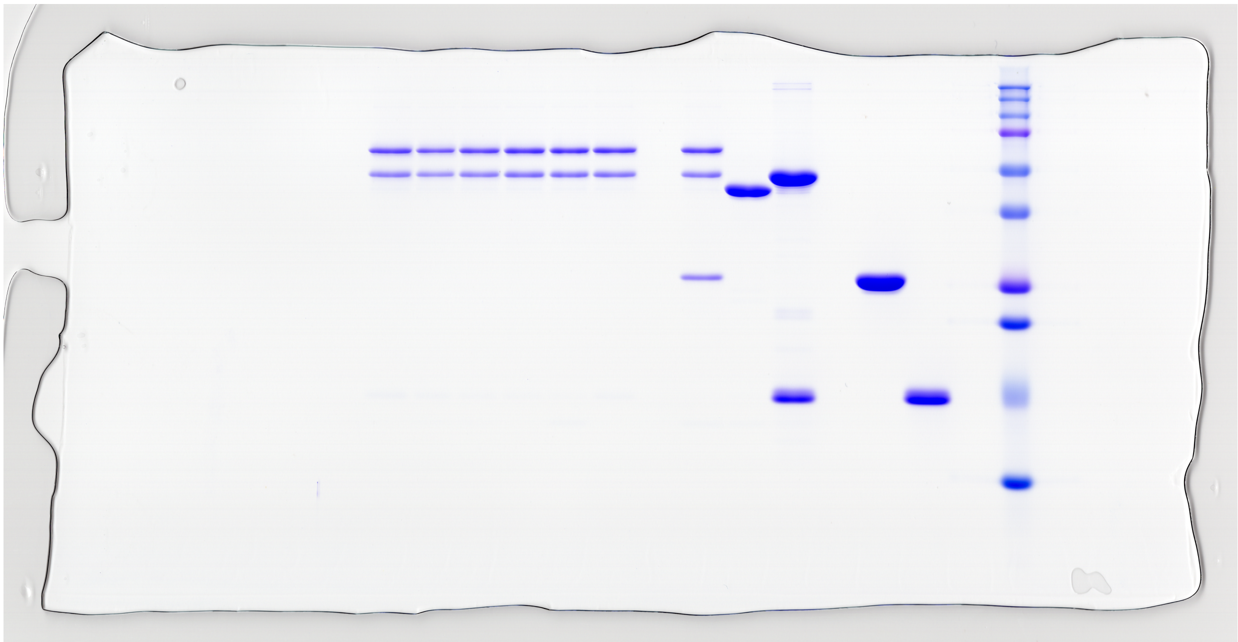

Source Data for Supplementary Figure 4b

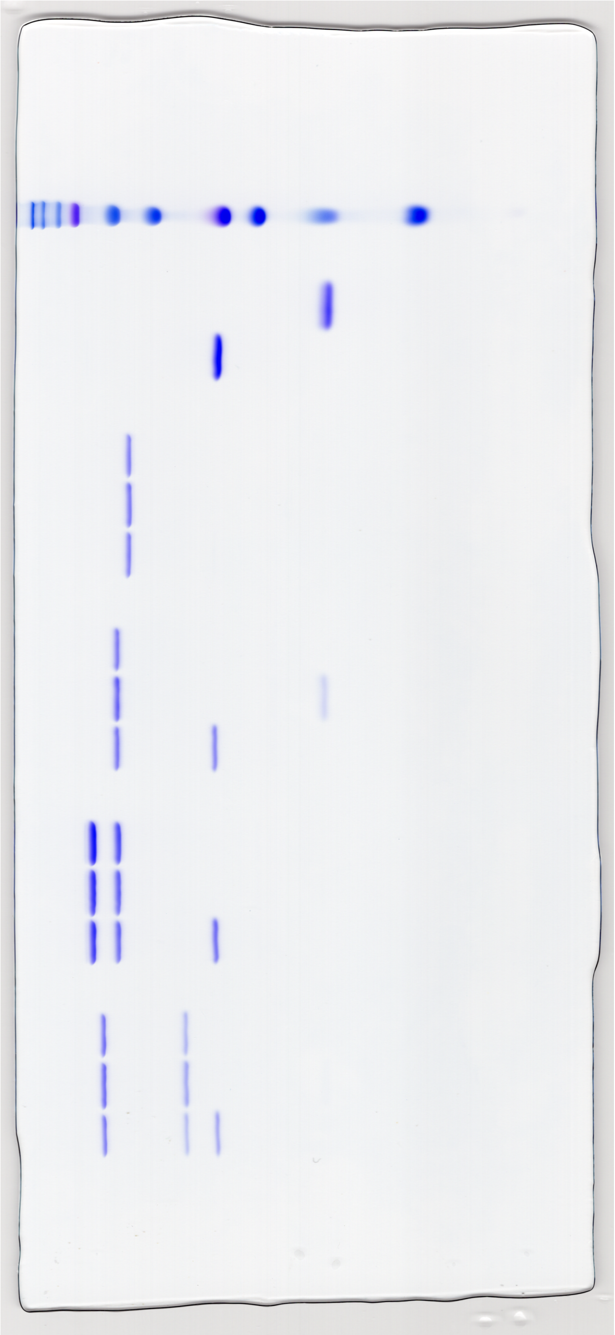

Source Data for Supplementary Figure 4c

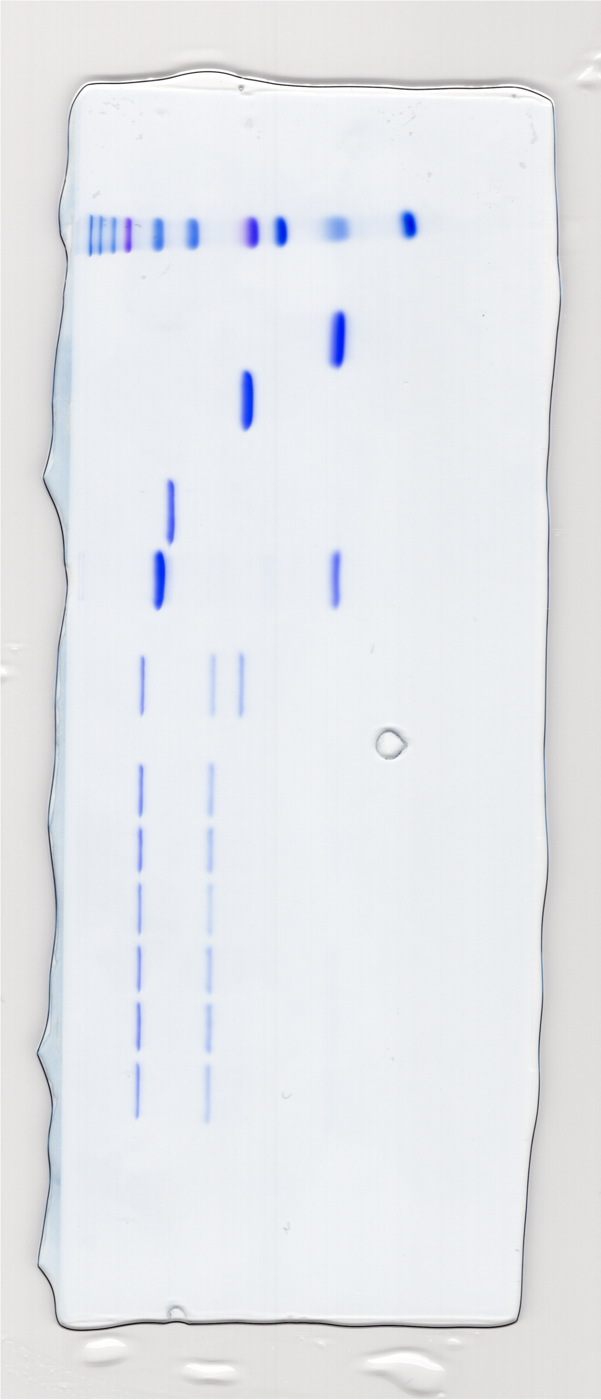

Source Data for Supplementary Figure 4d

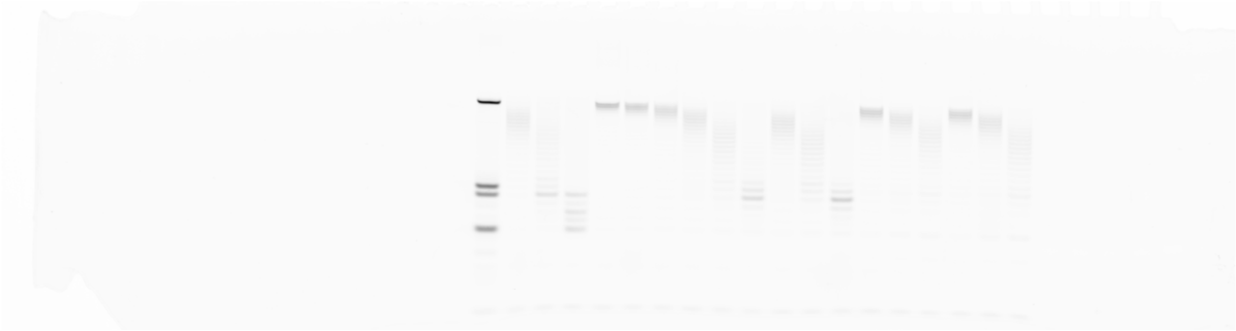

Source Data for Supplementary Figure 6b

t, min	NOT7+PieF_K124A		
8	18	18	18
16	15	15	15
32	10	10	10

t, min	NOT7+PieF_K124R		
8	20	20	20
16	19	19	19
32	16	17	16

t, min	NOT7+PieF_5M		
8	16	16	16
16	11	11	11
32	0	0	0

t, min	NOT7+PieF_2M		
8	18	19	18
16	15	17	15
32	10	12	10

t, min	NOT7+PieF_3M		
8	15	15	16
16	10	10	11
32	0	0	0

Source Data for Supplementary Figure 6h

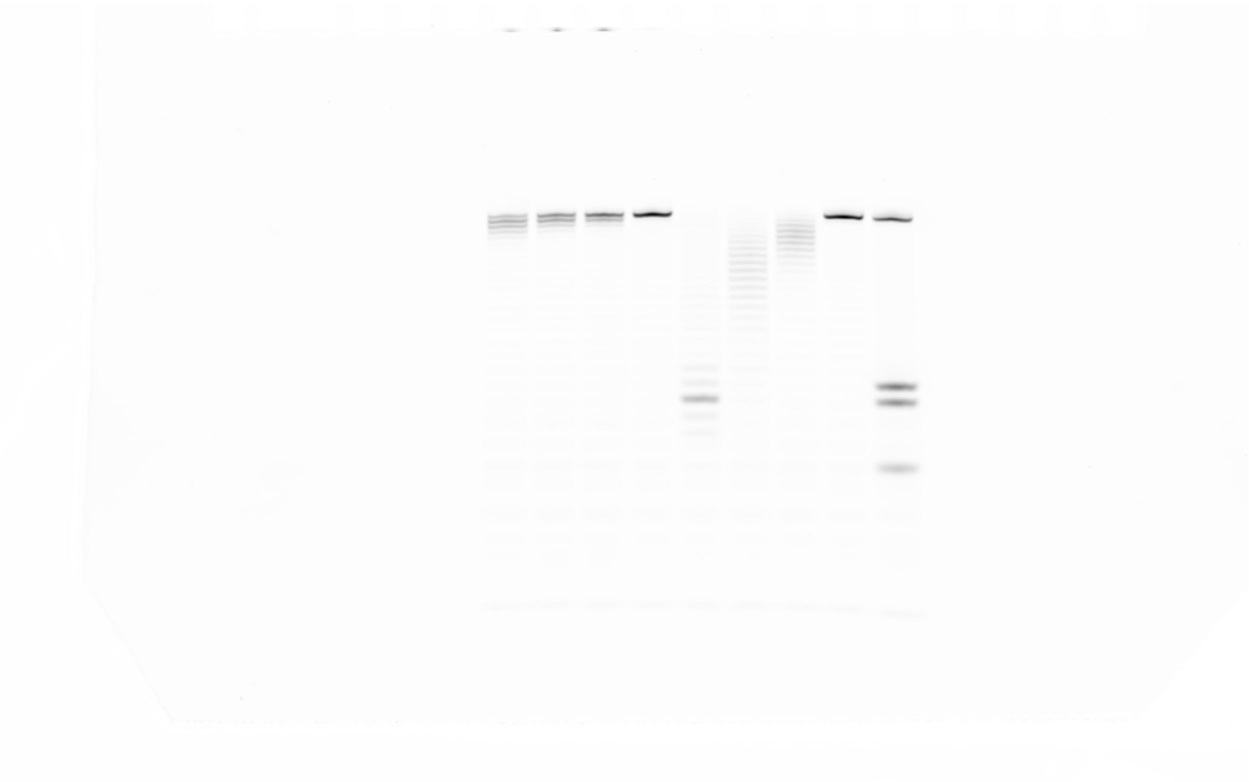

Source Data for Supplementary Figure 7a

Day 2			
GFP	PieF	5M	D40A
89.1	94.8	86.9	89.2
92.4	93.1	92.6	90.5
82.9	79.6	87	88.2
86.2	84.5	89.4	91

Day 6			
GFP	PieF	5M	D40A
93.4	77.6	93.3	70.2
97.1	78.5	94.9	77.4
92.2	82.9	95.9	85
94.7	92.4	89.5	88.3
85.5	89.5	89.7	85.4
87.2	87.7	92.4	88.8
90.5	85.8	88.7	89.6
89.1	86.1	94.1	87.5

Source Data for Supplementary Figure 7c

	G0/G1		S		G2	
	Mean	N	Mean	N	Mean	N
Vector	49.5	2	42.2	2	8.3	2
NOT7 D40A	39	2	51	2	10	2
PieF	38.9	2	53.2	2	7.9	2
PieF 5M	54.6	2	38.9	2	6.5	2
